# Increased Macular Pigment Optical Density and Visual Acuity following Consumption of a Buttermilk Drink Containing Lutein-Enriched Egg Yolks: A Randomized, Double-Blind, Placebo-Controlled Trial

**DOI:** 10.1155/2016/9035745

**Published:** 2016-03-14

**Authors:** Sanne M. van der Made, Elton R. Kelly, Aize Kijlstra, Jogchum Plat, Tos T. J. M. Berendschot

**Affiliations:** ^1^Department of Human Biology, NUTRIM School of Nutrition & Translational Research in Metabolism, Maastricht University Medical Centre, 6200 MD Maastricht, Netherlands; ^2^University Eye Clinic Maastricht, Maastricht University Medical Centre, 6202 AZ Maastricht, Netherlands

## Abstract

*Purpose*. To study the effect of 1-year daily consumption of a dairy drink containing lutein-enriched egg yolks on macular pigment optical density (MPOD) and visual function parameters in elderly subjects with ocular drusen and/or retinal pigment abnormalities.* Methods*. One hundred and one subjects were recruited to participate in this randomized, double-blind, placebo-controlled parallel intervention trial. Statistical analyses were performed with 46 subjects in the lutein group and 43 in the control group. MPOD, best corrected visual acuity (BCVA, logMAR), and dark adaptation were measured at the start of the study, after 6 months and after 12 months. Plasma lutein and zeaxanthin concentrations were assessed at baseline and at the end of the study.* Results*. In the lutein group, plasma lutein concentrations increased significantly from 205 ng/mL at baseline to 399 ng/mL after twelve months of intervention. MPOD increased significantly from 0.45 to 0.52 and BCVA improved significantly from −0.04 to −0.09 LogMar. Differences in rod dark adaptation rate between both groups were not significant.* Conclusion*. Daily consumption of a dairy drink containing lutein-enriched egg yolks for one year improves visual acuity, MPOD, and plasma lutein concentration in elderly subjects with drusen and/or retinal pigment epithelial abnormalities.

## 1. Introduction

Age-related macular degeneration (AMD) is the leading cause of irreversible blindness among the aged population in industrialized countries [1]. The incidence for late AMD varies from 1.4% in a Japanese study population >40 years [2] to 3.1% in a population aged 43–86 years in the US Beaver Dam Eye Study [3–5].

An increased intake of lutein via supplements has been shown to increase plasma lutein concentrations [6, 7] and to increase macular pigment level [8–10]. Although epidemiologic evidence evaluating the relation between dietary lutein and zeaxanthin intake and the risk for AMD is inconsistent [11, 12], ample evidence, including recent genetic data [13], points towards a protective effect of these carotenoids against AMD progression [14, 15]. Additionally, a study by Ma et al. [16] showed progressive improvements in macular pigment optical density (MPOD) and a trend towards improvement of best corrected visual acuity (BCVA) after lutein supplementation in early AMD patients.

Interestingly, bioavailability of lutein is shown to be two to three times higher from eggs than from spinach and lutein supplements, possibly caused by the lipid-rich matrix in which the lutein is provided [17]. From our previous study in one hundred healthy volunteers, it became clear that it is possible to significantly increase plasma lutein and zeaxanthin concentrations by twelve weeks consumption of a buttermilk drink that included lutein-enriched egg yolks [18]. MPOD did not change, which was most likely due to the relatively short intervention period. As described above, several studies have already shown an effect of lutein supplements on MPOD and visual function [19–22]; however, there are no reports yet that describe an effect of dietary lutein intake on visual function parameters. Therefore, the primary aim of this study was to assess the effects of 1-year daily consumption of a dairy drink containing lutein-rich egg yolks on MPOD and visual function parameters in elderly subjects with ocular drusen and/or retinal pigment abnormalities who had not yet been diagnosed with AMD.

## 2. Subjects and Methods

### 2.1. Subjects

One hundred and one subjects were recruited via advertisements in newspapers in the province of Limburg, the Netherlands, and 52 subjects were enrolled in the lutein group, while 49 subjects were enrolled in the control group. All participants gave their written informed consent before the screening procedure started. Eligible subjects were at least 50 years of age, showing drusen and/or retinal pigment epithelium alterations in at least one eye as evidenced by fundus photographs. Furthermore, visual acuity had to be >0.5, and eligible subjects should not have ocular media opacities, as assessed qualitatively by one of the staff ophthalmologists of our clinic, and were not allowed to use nutritional supplements containing lutein or zeaxanthin. Individuals willing to participate were excluded from taking part in the study when they used medication to treat diabetes, had cardiovascular diseases or disturbances in their lipid metabolism that demanded lipid-lowering treatment, or were allergic to eggs or egg products. One subject already withdrew after the screening and before the baseline visit. The study protocol was approved by the Medical Ethics Committee of Maastricht University Medical Centre and registered at ClinicalTrials.gov on May 14, 2009, as NCT00902408. All research and measurements followed the tenets of the Declaration of Helsinki and were performed within Maastricht University Medical Centre from October 30, 2009, through December 2, 2011.

### 2.2. Study Design

A one-year, randomized, double-blind, placebo-controlled intervention trial was conducted in elderly subjects with ocular drusen and/or retinal pigment abnormalities. Subjects were allocated to the control or lutein group according to a preestablished, computer generated randomization scheme. Allocation was concealed in sequentially numbered, sealed envelopes and stored by the study coordinator. Participants who were allocated to the experimental (lutein) group were asked to consume one lutein-enriched egg-yolk containing dairy drink daily (NWT-02, provided by Newtricious R&D, Oirlo, The Netherlands). The one and a half egg yolks in this drink were enriched in lutein, zeaxanthin, and DHA via the feed of laying hens and were incorporated in 80 mL buttermilk drink. The intervention products contained on average 1.38 ± 0.16 mg of lutein, 0.21 ± 0.02 mg of zeaxanthin, and 160 ± 10 mg of DHA. Subjects who were assigned to the control group received a similar buttermilk drink without the addition of 1.5 egg yolks. In order to keep participants and the study team unaware of treatment groups, the color of the control drink was matched to that of the lutein-enriched drink by adding synthetic colorants E104 and E110. Lutein, zeaxanthin, and DHA concentration were below detection limit in the control group. For one year, participants received a fresh delivery of buttermilk drinks at home every two weeks. These drinks were provided in 100 mL flasks that were packaged in carton boxes. Both flasks and boxes were coded with the randomization number of the subject.

### 2.3. Fundus Photography, Grading, and Classification

After maximal pupil dilatation was achieved using tropicamide 0.5% eye drops, fundus photographs were obtained using a Topcon TRC-50EX camera. Acquired images were centered on the fovea (1840 × 1224 pixels) and subtended 45°.

After the study finished, detailed grading for age-related maculopathy and age-related macular degeneration was performed on the fundus photographs taken from the test eye at the baseline visit according to the international classification and grading system [23, 24]. The grading was performed by an independent site, for example, at the Rotterdam Study Center.  Table 1 shows the distribution of AMD at baseline.

### 2.4. Macular Pigment Optical Density (MPOD)

MPOD was determined by heterochromatic flicker photometry (QuantifEYE; Topcon, Newbury, UK). In this device there are two light emitting diodes (blue, 470 nm, and green, 540 nm) that make up a target that flickers in counterphase. At the start of the test, the temporal flicker frequency is above the normal critical flicker fusion frequency (60 Hz) and is reduced at 6 Hz/sec. The subject fixates on the target and presses a button when flicker is detected. The luminance ratio of blue and green is then changed, incrementing blue and decrementing green. The temporal frequency is reset to 60 Hz and again ramped down at 6 Hz/sec, until the subject detects flicker and presses the response button. Starting with a green luminance being higher than the blue luminance, this cycle continues for a series of blue-green luminance ratios until a V shaped function is obtained with a clear minimum that corresponds to the equalization of the blue and green luminance. This process of detecting flicker for a series of blue-green luminance ratios is then repeated for peripheral viewing at 6 degrees eccentricity, where again a V shaped curve is obtained which provides a minimum for the periphery. From these two curves, the MPOD is calculated according to the formula MPOD = log⁡[*L*_*c*_/*L*_*p*_], where *L*_*c*_ and *L*_*p*_ are the luminance of the blue light at the minimum for central and peripheral viewing, respectively.

### 2.5. Visual Acuity

Best corrected visual acuity (BCVA) was measured with an internally illuminated Early Treatment Diabetic Retinopathy Study (ETDRS) logMAR chart at 4 m. Illumination of the testing room was set at 200 lux. The luminance of the charts was checked with a photometer (PR-650 SpectraScan Colorimeter). Mean luminance of the center of the charts was 180 cd/m^2^. Participants were asked to read all the letters they could recognize, monocularly with the testing eye, starting from the top left letter in the first row.

### 2.6. Dark Adaptation

Dark adaptation (DA) was measured monocularly with an undilated pupil. Prior to the beginning of a DA test, the tested eye was bleached with an electronic Flash Gun. The other eye was covered with an eye patch. Testing distance was set to 100 cm. Stimuli generated with a VSG 2/5 card (Cambridge Research Systems Ltd., Rochester, UK) were displayed on a Sony GDM-f500 high resolution graphics display. Neutral density filters were placed in front of the screen during the time-course of the test in order to increase its luminance range. This was achieved by covering the monitor screen with a 1.3 log unit neutral density filter for the first cone stage and subsequently adding 2 other filters each of 1.3 using a sliding mechanism at different stages as sensitivity improved. Following bleaching, the subjects fixated a red fixation target (0.3 degrees) presented at 11 degrees visual angle from the testing stimulus. The testing stimulus was composed of a white illuminant C (CIE 1931 coordinates are *x* = 0.31 and *y* = 0.316) 1 degree, temporally modulated (1 Hz) stimulus. Thresholds were determined with the method of adjustment. This procedure continued at approximately 2-minute intervals until an absolute threshold was reached.

Dark adaptation data were processed using a 7-parameter model using the Nelder-Mead method implemented in MATLAB (Nantick, MA). A text file was produced to include the 7 parameters of the model fit. These data were subject to the statistical analysis. Nonrational data such as excessive sensitivity values (e.g., 3000 dB) were removed by filtering so that the sensitivity range fell within 0–200 dB. The model was reapplied to this modified data set.

### 2.7. Plasma Lutein and Zeaxanthin Concentrations

Fasting blood samples were taken at the start of the study (T0) and after 12 months of intervention (T12), to assess plasma lutein concentrations. Blood samples were taken from a forearm vein after an overnight fast (no food or drink after 8 PM, except for water), by the same person, and at the same location. Plasma was obtained by sampling blood into EDTA tubes (Becton, Dickinson and Company, Franklin Lakes, NY, USA), followed by low-speed centrifugation at 1300 ×g for 15 min at 4°C.

Lutein and zeaxanthin concentrations were analyzed using high performance liquid chromatography (HPLC), as previously described [25]. Briefly, on the day of analysis, the samples were thawed and mixed well. Samples were deproteinized by adding 500 *μ*L sample to 500 *μ*L ethanol. After this, the samples were mixed and allowed to stand for 15 minutes at room temperature to complete precipitation of proteins. Subsequently, carotenoids were extracted by adding 1.0 mL n-hexane. After centrifugation for 10 minutes at 4°C and 3.000 ×g, 0.5 mL of the upper hexane layer was evaporated to dryness under a stream of nitrogen. The residue was dissolved in 0.5 mL of a mixture of methanol, acetonitrile (1 : 1), and dichloromethane and analyzed by HPLC. Separation was obtained on a C18 reversed-phase column, thermostatically controlled at 30°C. The samples were eluted by use of a mobile phase consisting of methanol, acetonitrile, 2-propanol, and water at a flow rate of 1.5 mL/min. Detection was performed with a diode array UV detector at 450 nm. Quantification was carried out by including commercially available lutein and zeaxanthin as a standard (Sigma-Aldrich, St. Louis, USA).

### 2.8. Statistical Analyses

Statistical analyses were performed using SPSS 18.0 (SPSS Inc., Chicago, IL, USA). Differences in gender distribution and smoking status over the experimental groups were tested using the Pearson Chi-square test, while baseline differences in age, plasma lutein and zeaxanthin concentrations, MPOD, and visual acuity were evaluated by an unpaired Student's* t*-test. A Linear Mixed Models (LMM) analysis with subject ID as grouping factor and diet and time and their interaction term as covariate was performed to evaluate differences in MPOD and VA. The same approach was used to evaluate differences within the control and lutein group regarding changes in MPOD during the 1-year intervention. Changes in plasma lutein and zeaxanthin concentrations over time were evaluated using an unpaired Student's* t*-test. *P* values were considered significant if *P* < 0.05. Results are shown as mean ± standard deviation (SD).

The expected increase in MPOD in our, earlier reported [18], one-year trial was estimated at 12%. Considering an MPOD measuring error of 17%, a significance level (*α*) of 5%, a power of 90%, and a 10% dropout rate, 48 subjects had to be included in the study. However, for one of the secondary outcome parameters in this study (reported elsewhere), which was flow-mediated dilation (FMD), 50 subjects should be included in both intervention groups to detect a true difference in FMD of at least 1%, assuming a standard deviation of FMD of 1.7%, a dropout rate of 10%, a power of 80%, and a significance level (*α*) of 5%. Therefore, a total of 101 subjects started the study which means that it was well powered to show significant effects for the measurements described here.

## 3. Results

### 3.1. Subject Characteristics

**Figure 1:**
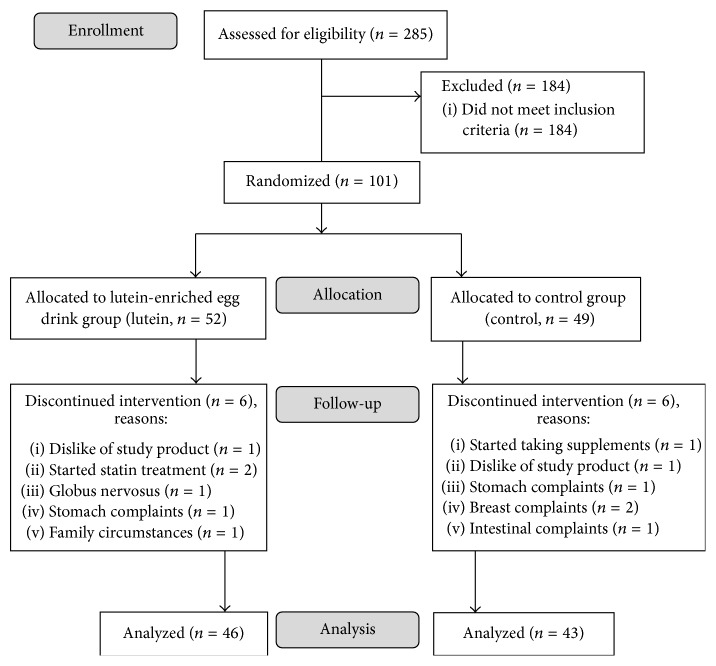
Subject flow chart.

During the 1-year follow-up of the study, twelve participants withdrew. The flow of participants throughout the study and reasons for discontinuation are shown in Figure 1. At baseline, no statistically significant differences were found between the lutein and control groups regarding age, BMI, plasma lutein concentration, MPOD, visual acuity, and dark adaptation (Table 2).

### 3.2. Macular Pigment, Visual Acuity, Dark Adaptation, and Plasma Lutein and Zeaxanthin

**Figure 2:**
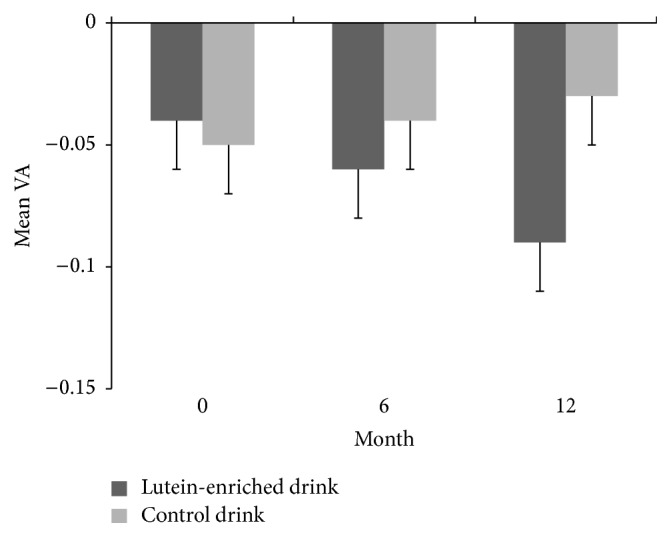
Mean (±SE) visual acuity (LogMAR) in time for the lutein group (dark grey) and the placebo group (light grey). Change in lutein group was significantly different from change in control group (*P* < 0.01).

Visual acuity improved upon receiving a lutein-enriched diet (*P* < 0.01, Figure 2). A significant decrease of 0.0052 ± 0.0017 LogMAR units per month (i.e., half a line increase on ETDRS chart over one year) was observed in the lutein group as compared to the control group (*P* < 0.01).

Although the rod dark adaptation rate showed a tendency to increase in the lutein group, which represents faster recovery, and a decrease in the placebo group (i.e., a further slowing down of the rod dark adaptation rate), differences between the groups were not significant following statistical analysis (*P* = 0.14; data not shown).

**Figure 3:**
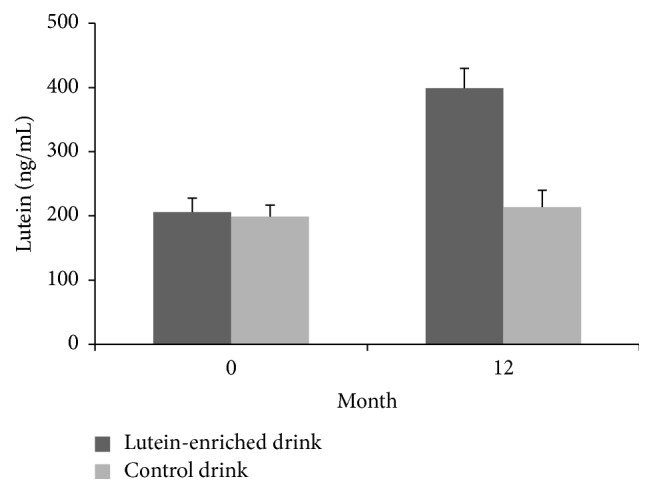
Mean (±SE) plasma lutein concentration in time for the lutein group (dark grey) and the placebo group (light grey). The increase in the lutein group was significantly different from the change in the control group (*P* < 0.001).

**Figure 4:**
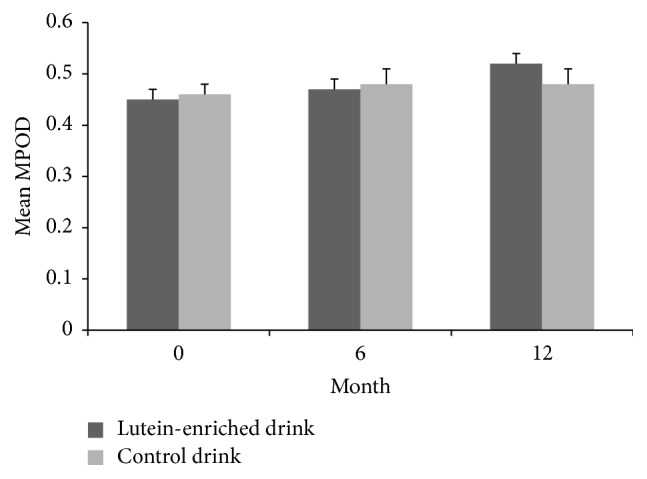
Mean ± SE macular pigment optical density (MPOD) in time for the lutein group (dark grey bars) and the placebo group (light grey bars). MPOD increased significantly in the lutein group as compared to the control group (*P* < 0.05).

We observed a significant 94% increase in plasma lutein concentrations from 205 to 399 ng/mL in the lutein group and no significant change in the control group (*P* < 0.001, Figure 3). A similar increase in plasma zeaxanthin was observed in the lutein group (*P* < 0.01). Consequently, also MPOD increased significantly in the lutein group as compared to the control group (*P* < 0.05, Figure 4) and within the lutein group as compared to baseline (Table 3). The increase in MPOD in the lutein group compared to the control group was on average 0.0041 ± 0.0019 per month.

## 4. Discussion

This study shows that it is possible to increase plasma lutein and improve visual acuity and macular pigment optical density by daily consumption of a dairy drink containing lutein-rich egg yolks in elderly subjects with drusen and/or retinal pigment abnormalities. To the best of our knowledge, the effect of long term consumption of a functional food on visual function parameters has not yet been reported earlier. The individuals included in our study had ocular drusen and/or retinal pigment abnormalities in at least one eye, but they had not yet been diagnosed with AMD at the time of recruitment. Analysis of the fundus photographs by an independent referral center showed that 48% of the eyes would have been classified as AMD grades 1 to 4. In view of the small numbers we did not perform a subgroup analysis concerning MPOD or BCVA response.

Following the diet with a lutein-enriched egg drink (1.4 mg extra lutein/day), we observed an increase in MPOD concentrations after 12 months. An earlier pilot study from our group did not show an effect of a lutein-enriched egg diet on MPOD levels in healthy subjects. Besides the different study population, this is probably due to the shorter intervention period of 3 months of this previous study [18]. A study by Murray et al. [6] showed a significant increase in MPOD after twelve-month intake of 10 mg lutein ester. In this study, the increase in MPOD relative to the control group was 39.5%, which is considerably higher than the 10.7% increase observed in our study. However, in the latter study, the group supplemented with lutein had a significantly lower baseline MPOD than the placebo group [6]. Others showed an increase in MPOD in two lutein-supplemented groups that either received a daily 10 or 20 mg dose for 48 weeks [7]. Unfortunately, no comparison was made between intervention groups and placebo group, which makes it hard to draw conclusions on the true effect of lutein on MPOD in this particular study. A one-year study in subjects with early AMD showed an increase in MPOD following intake of one out of three combinations of lutein, zeaxanthin, and* meso-*zeaxanthin [26]. However, no placebo group was included in this study. Another study [27] found a 27.2% relative increase in MPOD after the daily intake of 20 mg lutein for three months followed by 10 mg daily for a time period of three months. In this study, a spectroscopic technique was used to measure MPOD, whereas in our study, and in most other studies until now, MPOD is assessed by flicker photometry [28–30]. A trial describing a twelve-month lutein supplementation in atrophic AMD patients reported a significant 36% MPOD increase in the lutein group, whereas a decrease in MPOD was observed in the control group [19]. Furthermore, a recent meta-analysis concluded that dietary supplementation of lutein leads to a significant improvement in MPOD [22]. Although the increase in MPOD as seen in our study was significant, it did not reach the relative increase as observed in earlier studies using lutein containing pill supplements. The reasons for this discrepancy are not clear but could be associated with the high baseline MPOD of the subjects in our study as compared to the other studies [6, 19, 27, 31].

Macular pigment is composed of three different carotenoids including lutein, zeaxanthin, and* meso*-zeaxanthin, whereby lutein is mainly found in the peripheral macula, whereas zeaxanthin and* meso*-zeaxanthin are present in the center. Eggs are a well-known source of both lutein and zeaxanthin and after consumption they may accumulate in the retina. Retinal* meso*-zeaxanthin will be incorporated in the macula following local bioconversion from lutein or may be acquired via other foodstuffs.

While the increase in MPOD was not as pronounced as shown in the supplement studies mentioned above, we did show a significant improvement over time in visual acuity in the lutein intervention group as compared to the control group. Our data are in agreement with earlier studies showing an increase in both MPOD and VA after twelve months of daily 10 mg nonesterified lutein or 10 mg lutein combined with a range of antioxidants and vitamins [19]. Visual acuity did not improve in the study by Murray et al. [6], which was argued to be caused by the fact that >50% of the population already had a normal or above normal VA. Indeed, in their study, a significant improvement in VA was found in a subpopulation of subjects with a low VA at baseline. Additionally, a recent meta-analysis showed a significant improvement in VA after lutein and zeaxanthin supplementation in four out of seven studies. A slightly stronger effect was found in studies with higher-dose (20 mg lutein daily) supplementation [32]. Although the amount of lutein given in our study is considerably lower than the dosages given in these studies, we found a significant improvement in VA. It should be noted that the lutein group in our study started with a relatively good VA of −0.04 logMAR. Despite the adequate VA in these subjects, we still observed a small but statistically significant improvement after the one-year intervention. This implies that the effect in our study might have even been more pronounced if we would have included subjects with a lower baseline VA. Additionally, this meta-analysis found a dose-dependent improvement in contrast sensitivity after lutein supplementation [32], which indicates that this parameter should be further explored in the target population as performing additional visual function tests to detect subtle changes in the macula is clearly important.

The quantity of lutein used in the current study (1.4 mg/day) was less than the amount used in studies that provided lutein capsules, which varied between 5 and 20 mg of lutein [33]. Still, we were able to show a significant increase in plasma lutein concentrations in the intervention group over the one-year course after consuming the lutein-enriched egg yolks that provided an average additional daily lutein intake of 1.4 mg. This underlines the finding that lutein has a high bioavailability from eggs [17], which is most probably caused by the matrix in which the lutein is incorporated [34]. Lutein bioavailability after taking a capsule supplement is dependent on the quantity of fat in the meal during which it is taken [35].

Several studies have evaluated the effect of lutein supplementation on plasma lutein concentration. A study with university students taking 10 mg lutein and 2 mg zeaxanthin over a time period of 300 days showed a 400% increase in plasma lutein concentration [36]. Other studies showed significant increases in serum lutein concentration varying from 17 to 555% change in both normal and AMD subjects after only 8 weeks intake of one out of three combinations of lutein, zeaxanthin, and* meso*-zeaxanthin [37]. These results were reproduced in a three-year study using the same intervention in forty-seven subjects with early AMD [38]. However, it must be noted that no placebo arm was included in both studies.

As reflected by the high standard deviation of plasma lutein concentrations in our study, it is clear that there is a marked difference in the individual response to lutein supplementation. Genetic polymorphisms are suggested to play a role in bioavailability of lutein and zeaxanthin and influence both serum and retina status of these carotenoids. In a recent study by Meyers and colleagues, these genetic determinants were found to be independent of dietary lutein and zeaxanthin intake [13]. This study showed several genes to be associated with serum lutein and zeaxanthin concentration, including stAR-related lipid transfer protein 3 (*STARD3*), which is involved in xanthophyll binding in the retina; ATP-binding cassette subfamily G member 8 (*ABCG8*); and cholesteryl ester transfer protein (*CETP*), which are involved in high-density lipoprotein (HDL) transport [13]. Another study also showed a high interindividual variability (CV 75%) in postprandial chylomicron lutein response after consuming lutein-enriched meals, which was found to be genetically determined [39]. Furthermore, there might be an optimum in terms of lutein and/or zeaxanthin supplementation. It was hypothesized that there is duodenal, hepatic-lipoprotein, or retinal carotenoid competition for carotenoid uptake [40]. These findings suggest that future research into the relation between dietary lutein and improving visual performance should also take genetic background of the subjects into account.

Epidemiological and intervention studies indicate that a higher lutein intake may delay AMD development [12]. Increased consumption of dietary cholesterol from lutein-enriched eggs might lead to raised serum cholesterol concentrations [41], which in turn is associated with a higher risk of developing cardiovascular disease (CVD). However, a recent meta-analysis revealed that a higher consumption of eggs (up to one egg per day) was not associated with increased risk of coronary heart disease or stroke [42]. We assessed the possible side effects of our trial and showed that the consumption of the lutein-enriched egg-yolk containing buttermilk drink daily for a time period of 1 year did not lead to a significant increase of serum total, HDL, and LDL cholesterol, as well as the ratio of total cholesterol to HDL cholesterol [43].

In conclusion, we here show that daily consumption of a lutein-enriched egg drink for one year leads to a significant increase in visual acuity and is capable of enhancing both plasma and macular concentrations of lutein.

## Figures and Tables

**Table 1:** Distribution of AMD at baseline in the present study.

Grade	Criteria	Lutein group (*n* = 51)	Placebo group (*n* = 49)
0	Any small drusen	26	24
1	Soft distinct drusen	15	7
2	Indistinct drusen	5	8
3	Soft drusen/pigment changes	2	8
4	Atrophic changes	1	1
Total		49	48

**Table 2:** Baseline visual acuity (VA), macular pigment optical density (MPOD), and plasma lutein concentrations in lutein and control groups.

	Lutein group (*n* = 51)	Control group (*n* = 49)	*P* value
Gender (m/f)	17/34	15/34	0.832
Age (years)	62 ± 7	63 ± 8	0.557
BMI (kg/m^2^)	26.7 ± 3.6	26.0 ± 3.8	0.375
Ever smoked (yes)	24	20	0.472
Lutein (ng/mL)	206 ± 148	199 ± 118	0.803
MPOD	0.45 ± 0.14	0.46 ± 0.16	0.859
VA (LogMar)	−0.04 ± 0.14	−0.05 ± 0.13	0.711
Dark adaptation	−0.17 ± 0.05	−0.19 ± 0.06	0.302

MPOD, macular pigment optical density; VA, visual acuity.

**Table 3:** Mean changes in MPOD in lutein and control groups during the time course of the study.

	Mean MPOD	Absolute change from baseline	SE	% change from baseline	*P* value
Lutein group					
Baseline, *n* = 46	0.45	—	0.02	—	—
6th month, *n* = 41	0.47	0.05	0.02	4.4	<0.001
12th month, *n* = 45	0.52	0.07	0.02	15.6	<0.001
Control group					
Baseline, *n* = 46	0.46	—	0.02	—	—
6th month, *n* = 38	0.48	0.02	0.03	6.7	0.18
12th month, *n* = 43	0.48	0.02	0.03	4.4	0.34

A linear mixed model approach was used to assess differences.
